# Supplementary material for: Genome-Wide Identification and Characterization of the VQ Motif-Containing Gene Family Based on Their Evolution and Expression Analysis under Abiotic Stress and Hormone Treatments in Foxtail Millet (Setaria italica L.)
Source: Genes (Basel). 2023 Apr 30;14(5):1032. doi: 10.3390/genes14051032 (PMC10218184; doi:10.3390/genes14051032)
Supplement: Supplementary file 1 [file genes-14-01032-s001.zip › Supplementary Figure.pdf]

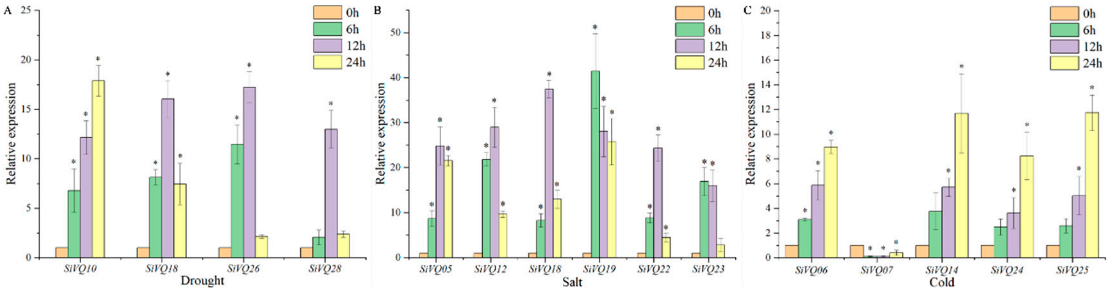

Figure S1. Expression analysis of significantly induced *SiVQ* genes under abiotic stress based on qRT-PCR. A PEG 6000, B NaCl, C 4℃.

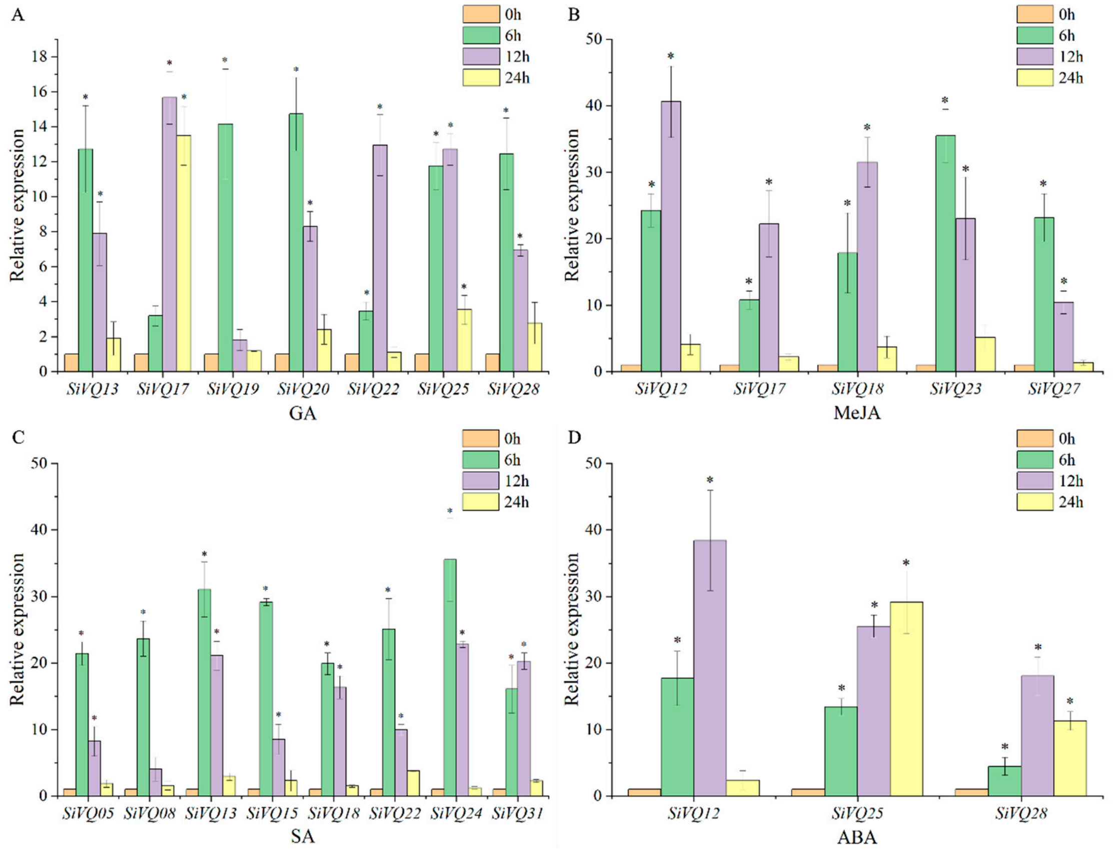

Figure S2. Expression analysis of significantly induced *SiVQ* genes under hormone treatments based on qRT-PCR. A Gibberellin (GA), B Methyl jasmonate (MeJA), C Salicylic acid (SA) and D Abscisic acid (ABA).
